# How Does Manufacturing Agglomeration Affect Green Development? A Spatial and Nonlinear Perspective

**DOI:** 10.3390/ijerph191610404

**Published:** 2022-08-21

**Authors:** Huaxi Yuan, Longhui Zou, Xiangyong Luo, Yidai Feng

**Affiliations:** 1 Institute of Industrial Economics, Chinese Academy of Social Sciences, Beijing 100006, China; 2 School of Economics, Zhongnan University of Economics and Law, Wuhan 430073, China; 3 Department of Modern & Classical Language Studies, Kent State University, Kent, OH 44240, USA; 4 Department of Civil and Environmental Engineering, University of Wisconsin-Milwaukee, Milwaukee, WI 53211, USA; 5 School of Management, Wuhan Institute of Technology, Wuhan 430205, China

**Keywords:** manufacturing agglomeration, green development, GS2SLS, mediating effect, Yangtze River Economic Belt

## Abstract

Developing high-quality manufacturing industries and realizing green transformation are relatively pressing issues in the 21st century. Existing studies only focus on the economic or environmental effects of agglomeration, and the combined effects of manufacturing agglomeration have been neglected. Therefore, by referring to industrial agglomeration theory and constructing a theoretical analytical framework for manufacturing agglomeration and green development, this paper adopts the spatial panel Durbin model and mediating effect model with the panel data from China’s Yangtze River Economic Belt to empirically test the influence and its mechanism of manufacturing agglomeration on green development. The results show that: (1) There are significant temporal and spatial differences in green development in the Yangtze River Economic Belt. Overall, green development has maintained a steady increase on the timeline, but each region shows a hierarchical structure of “multiple peaks-multiple centers”. (2) There is a typical inverted U-shaped relationship between manufacturing agglomeration and green development, and the linear and quadratic coefficients of manufacturing agglomeration are −0.585 and −0.167, respectively. (3) Under the constraints of temporal, spatial, and urban heterogeneity, the impacts of manufacturing agglomeration on green development show significant differences. (4) Manufacturing agglomeration affects green development through three paths: the labor force upgrading effect, industrial structure upgrading effect, and technological innovation effect. The study can provide a theoretical and empirical basis for the green development of developing countries around the world.

## 1. Introduction

Since the inception of the reform and opening-up process, China has been developing its economy at an average annual rate of 9.7% and growing into the world’s second-largest economy [1]. From 1970 to 2017, China’s GDP increased from $149.5 billion to $12.24 trillion, lifting 800 million people out of poverty and entering the ranks of upper-middle-income countries [2]. However, this development model that relies heavily on energy consumption at the expense of the environment has also brought huge economic and social problems to China. In 2019, China’s total carbon dioxide emissions accounted for approximately 28.76% of the world’s total emissions [3], making it the world’s largest carbon emitter for 15 consecutive years [4]. China ranked 120th out of 180 countries and regions in the Environmental Performance Index in 2019. According to Xie et al. [5], if air pollution is not controlled strictly, health and economic impacts from PM_2.5_ and O_3_ will account for 2% and 0.09% of China’s GDP, respectively. Current and potential environmental pollution problems are threatening the sustainable development of the entire Chinese society. In view of this, the Chinese government is committed to promoting the theory and practice of green development by actively responding to the global green economy initiatives [6]. The coordinated development of economic growth and environmental protection has always been the focus of green development, which aims at achieving sustainable development within the carrying capacity of the ecological environment [7].

Manufacturing agglomeration (MA) is a main factor influencing green development (GD) [8]. It contributes 32% to China’s GDP and supplies 12% of the world goods exports from China [9], causing 67.9% of the energy consumption and 83.1% of the carbon dioxide emissions in total [10]. To minimize the negative externalities brought about by MA and to promote green development, in 2021, the Chinese government clearly stated in the “Fourteenth Five-Year Plan for National Economic and Social Development (2021–2025)” (http://www.gov.cn/xinwen/2021-03/14/content_5592884.htm, accessed on 18 January 2022) that it is necessary to promote the deep integration of Internet, big data, and artificial intelligence, and to promote the agglomeration of advanced manufacturing industries. Generally speaking, more than 70% of the manufacturing companies in China are gathered in three major metropolitan areas, including the Yangtze River Delta, Pearl River Delta, and Beijing–Tianjin–Hebei region. These regions have become the largest sources of energy consumption and pollutant emissions [11]. The above analysis leads to the following question: how does MA affect GD? This article attempts to conduct an in-depth investigation on the relationship between MA and GD from both theoretical and empirical aspects.

Compared with the existing research, the contribution of this research is mainly reflected in the following three aspects: (1) This article constructs a theoretical framework between MA and GD, and analyzes the interaction and mechanism between the two based on the industrial agglomeration theory and the reality of China. The existing research mostly discusses the role of MA on economic growth or environmental quality on the basis of the externality theory, but seldom analyzes the comprehensive influence of MA on GD. (2) In order to overcome the possible regression bias caused by the spatial spillover effect and endogeneity, this paper adopts the spatial panel Durbin model, instrumental variables, and the generalized spatial two-stage least squares (GS2SLS) method to analyze the influence of MA on GD. This is helpful to improve the accuracy of conclusions, which can provide an empirical basis for global policies on green development transformation. To this end, this paper selects whether the trade ports opened in Qing dynasty in 1842–1909, whether there was a railway passing by in 1933, and relief amplitude as instrumental variables (IV) for MA. Previous studies often neglect the endogenous problems and spatial spillover effects of agglomeration; hence, biased estimation results are usually obtained. (3) Through multidimensional heterogeneity analysis and empirical analysis of the influence mechanism, the differential impacts of MA on GD and the mechanism under multiple constraints of heterogeneity are explored, which can provide suggestions for targeted policy recommendations for sustainable development and green transformation. Most studies are limited to the impact of MA on macro factors, but fail to consider the impact of temporal, spatial, and urban heterogeneity on the relationship between the two, and rarely pay attention to the influence mechanism of MA on GD.

## 2. Literature Review

MA has always been a key issue in the research of industrial economics, new economic geography, and other disciplines. Since the late 1980s and early 1990s, many scholars have conducted in-depth research on the causes, mechanisms, effects, and theoretical frameworks of MA [12,13,14]. Some scholars have also noticed that MA influences macro-level economic factors through externalities, including economic growth, industrial structure upgrading, and innovation [15,16,17,18,19].

Current research on MA mainly focuses on its impact on environmental quality. Li et al. (2021) used the spatial panel regression model to test the relationship between industrial agglomeration and haze pollution and found that industrial agglomeration would aggravate haze pollution in local and neighboring areas [20]. This conclusion is also applicable to the samples from the provinces of China and the Bohai Sea Economic Region [21,22]. However, Fang et al. (2020) investigated the impact of MA on smog pollution based on the data of 283 Chinese cities between 2003–2013, and found that MA can significantly reduce smog pollution [23], which is in line with Huang et al. (2021)’s conclusions [24]. Furthermore, some studies found that MA may have an inverted U-shaped curve relationship with environmental quality [22,25].

In addition, some scholars also mention that there might be a reverse causality between MA and environmental quality, which leads to biased regression coefficients. Wu et al. (2021) used 134 Chinese cities as historically famous cities during the period of 1982–2018 to alleviate the possible endogenous problems of MA [26]. To overcome the endogeneity of MA, Chen et al. (2018) analyzed the relationship between industrial agglomeration and CO_2_ by using 110 trade ports that were forced to open in the Qing Dynasty between 1842–1909 [27]. Some scholars also argue that certain factors may cause MA to have a heterogeneous impact on environmental quality, such as differences in regional development, agglomeration modes, and industrial development [20,28,29].

In summary, although in-depth research has been carried out on the relationship between MA and economic development or environmental quality, there are still the following shortcomings: (1) Existing research only discusses the impact of MA on economic development or environment. The comprehensive impact of MA on economic development and environment is seldom evaluated. (2) Existing research rarely controls for spatial effects, nonlinear characteristics and endogenous issues of variables in a single model, resulting in biased regression results. (3) Existing research pays insufficient attention to the heterogeneity between MA and GD, which fails to reveal the mechanism between the two. Therefore, the research conclusions cannot help to optimize local practices.

## 3. Conceptual Framework

### 3.1. Direct Path of MA Affecting GD

**Figure 1:**
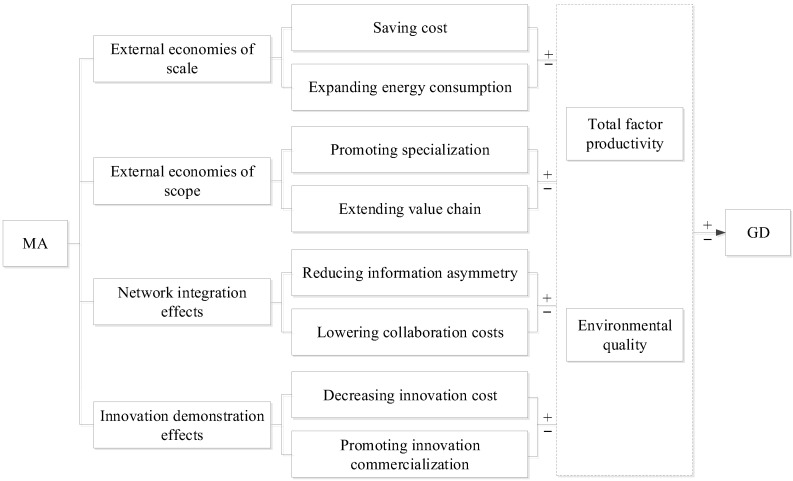
Direct path for MA to influence GD.

An important factor of MA that directly affects GD is the externality of agglomeration [30]. It is manifested in external economies of scale, external economies of scope, network integration effects, and innovation demonstration effects (Figure 1). Green development requires improving environmental quality while achieving green economic growth [31]. Therefore, when analyzing the interaction mechanism between MA and GD, we should focus on how MA affects total factor productivity and environmental quality.

External economies of scale of MA tend to impact GD in two ways. First, the new economic geography believes that MA can bring about significant scale economy effects [32]. That is, through spatial agglomeration, manufacturing industries can share the software and hardware facilities for production and operation in order to reduce their production costs and increase their total factor productivity. Specifically, on the one hand, enterprises in concentrated areas can share the infrastructure in the region, reduce the investment of fixed assets, and reduce production costs. On the other hand, because the external economies of scale help to reduce the marginal production costs, enterprises tend to further expand their production scale to increase their profit margins, which will inevitably lead to an increase in total energy consumption and pollutant emissions in the region [26]. This would reduce environmental quality.

External economies of scope of MA tend to impact GD in two ways. First, specialized MA can help to improve the level of regional specialization and labor division, and thus, encourages enterprises to give play to their respective production advantages to increase their total factor productivity [33]. Second, diversified MA is conducive to promoting upstream and downstream enterprise collaboration, extending the industrial value chain, and realizing regional vertical integration of production. This will improve total factor productivity in the region [34]. However, labor division and collaboration among enterprises are likely to prompt enterprises to continuously scale up production and pursue higher profits, leading to an increase in total regional energy consumption and total pollutant emissions, and ultimately, to the destruction of local ecosystems.

Network integration effects of MA tend to impact GD in two aspects. First, modern enterprises often compete in information technology. Establishing a network of MA is conducive for promoting the speed of information dissemination and reducing the degree of information asymmetries encountered by enterprises in the production and operation process [35]. The result is an increase in total factor productivity. Second, establishing a network of MA is conducive for forming a good and stable cooperative relationship and trust among enterprises. This reduces the tendency of opportunistic behavior in operating enterprises and the cost of contract execution and supervision [36].

Innovation demonstration effects of MA tend to impact GD in two ways. First, because of geographical proximity, enterprises and employees in concentrated areas can gain a great deal of innovative ideas, cases, and materials at a low cost or even for free. In this sense, they can build up rich innovative knowledge, reduce the costs of research and development [37], and thereby increase total factor productivity. Second, technological innovation is the core factor for maintaining the competitiveness of enterprises. Enterprises in concentrated areas can achieve faster product innovation, save on the “distance friction costs” of disseminating product innovation, and quickly apply this knowledge to further production and operations. The result is increased total factor productivity. However, innovation is a double-edged sword, which not only promotes the productivity of enterprises, but also stimulates the expansion of the production scale and increases energy consumption, thus damaging the environmental quality of the region.

### 3.2. Indirect Path of MA Affecting GD

**Figure 2:**
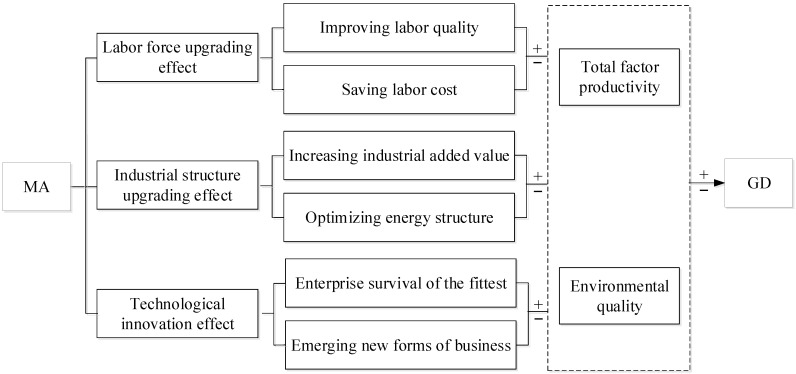
Indirect path of MA affecting GD.

According to the economic principle of agglomeration, three main sources of motivation for MA are the labor pool (“human”), intermediate input sharing (“object”), and knowledge overflow (“knowledge”) [38]. Based on this, this paper attempts to analyze how MA affects GD based on three aspects, i.e., the labor force upgrading effect, industrial structure upgrading effect, and technological innovation effect (Figure 2).

MA affects GD through the labor force upgrading effect. MA can promote labor concentration and alleviate the problem of labor force recruitment. It can help to improve the degree of matching between enterprises and employees, reduce the costs of recruitment, and thus, save labor costs. This will increase total factor productivity [39]. Although improvements in labor quality and savings in labor costs can significantly increase the productivity of enterprises, they may also lead to a sharp increase in energy consumption, thereby damaging the regional ecological environment.

In addition, MA affects GD through the industrial structure upgrading effect. MA can promote industrial structure upgrading [40], which facilitates the move from low-end to high-end manufacturing industries. Industrial structure upgrading can increase the added value of the industry, and thus, increase the total factor productivity of enterprises. It also promotes the transformation of the energy structure from a polluting type dominated by fossil energy to a clean type, thereby improving the regional environmental quality.

Furthermore, MA affects GD through the technological innovation effect. MA benefits enterprises through technological innovation [41]. Technological innovation can help enterprises in concentrated areas to win the survival-of-the-fittest contest, that is, it enables competitive, innovative enterprises to survive, and phases out backward enterprises in concentrated areas. Therefore, the competitiveness and total factor productivity of enterprises in the concentrated area is improved. Through technological integration, industrial restructuring, and value chain extension, technological innovation can bring about new business patterns and iterative upgrading of regional industrial structure. However, technological innovation can also be destructive [42]. Technological innovation in the manufacturing industry tends to increase enterprises’ production scale and capacity. This might further exacerbate energy consumption, resulting in an increase in pollutant emissions and a decrease in the improvement of GD.

## 4. Research Design

### 4.1. Methodology

#### 4.1.1. Spatial Panel Durbin Model

Miller and Upadhyay (2000) tested the impact of opening up, directional trading, and human capital on total factor productivity by building a model of endogenous total factor productivity [43]. However, this study believes that technological development is not only affected by exogenous technologies, but also determined by the degree of MA. Accordingly, the production function is expressed as follows:

(1) $$Y_{it}=A_{it}\left(\cdot \right)F\left(K,\;L,E\right)$$

where *i* is the city, *t* is the year, *Y* means GDP, *K* is capital investment, *L* represents labor force input, and *E* is energy input; $A_{it}\left(\cdot \right)$ represents the efficiency function of the Hicks-neutral technological advances. This paper examines the impact of MA on GD based on Equation (1). According to van Hulten et al. (2006) [44], the Hicks efficiency term $A_{it}\left(\cdot \right)$ and its components in Equation (2) are multicomposed, namely:

(2) $$A_{it}=g\left(MA_{it}\right)=A_{i0}\cdot e^{\alpha_{it}}\cdot MA_{LQ}{}_{it}^{\beta_{1}}$$

The formula for calculating GD is obtained by substituting Equation (2) for Equation (1) and dividing by F(K, L,E) on both sides:

(3) $$GDL_{it}=\frac{Y_{it}}{F\left(K,\;L,E\right)}=A_{i0}\cdot e^{\alpha_{it}}\cdot MA_{it}^{\beta_{1}}$$

As can be seen from Equation (3), GD is influenced not only by K, L,and E, but also by the influence of MA. Equation (4) is obtained by taking the natural logarithm on both sides of Equation (3):

(4) $$lnGDL_{it}=\alpha +\beta_{1}lnMA_{it}+\delta_{j}\sum^{\;}X_{it}+\mu_{it}$$

where a is the model parameter; $GDL_{it}$ indicates GD during *t* period in *i* city; $MA_{it}$ represents the extent of MA during *t* period in *i* city; $X_{it}$ is a series of control variables;$\;\beta_{1}$ and $\delta_{j}$ are parameters of explanatory variables and control variables; $u_{it}$ are random interference terms.

To examine the nonlinear effect of MA on GD, this study further integrates the quadratic terms of MA in the model:

(5) $$lnGDL_{it}=\alpha +\beta_{1}lnMA_{it}+\beta_{2}{\left(lnMA_{it}\right)}^{2}+\delta_{j}\sum^{\;}X_{it}+\mu_{it}$$

Based on Equation (5), this paper further incorporates the spatial effect, using the spatial panel Durbin model to examine the impact of MA on GD in the following formula:

(6) $$lnGDL_{it}=a+\rho \overset{n}{\underset{i=1}{\sum }}W_{it}lnGDL_{it}+\theta_{1}lnMA_{it}+\gamma_{1}\overset{n}{\underset{i=1}{\sum }}W_{it}lnMA_{it}+\theta_{2}{\left(lnMA_{it}\right)}^{2}\;+\gamma_{2}\overset{n}{\underset{i=1}{\sum }}W_{it}{\left(lnMA_{it}\right)}^{2}+\theta_{j}\overset{n}{\underset{i=1}{\sum }}lnX_{it}+\gamma_{j}\overset{n}{\underset{i=1}{\sum }}W_{it}lnX_{it}+\varepsilon_{it}$$

where ρ is the coefficient of spatial lagged terms; *W* is the spatial weight matrix of 110 × 110, and the inverse distance matrix is adopted here [45].The distance between cities is calculated by the cities’ latitude and longitude. Other variables are the same as above.

#### 4.1.2. Mediating Effect Model

For further clarification, according to Feng et al. [46], this paper uses the classic mediating effect model to investigate how MA affects GD. The testing procedures of the mediating model are as follows:

(7) $$Y_{it}=cX_{it}+e_{1}$$

(8) $$M_{it}=aX_{it}+e_{2}$$

(9) $$Y_{it}=c^{\prime }X_{it}+bM_{it}+e_{3}$$

where *Y* is the explained variable, which refers to GD. *M* is the mediating variable, which represents the industrial structure upgrading effect, labor force upgrading effect, and technological innovation effect. *X* is the explanatory variable, which refers to MA; *c* represents the total effect of MA on GD. *a* is the effect of MA on mediating variable *M*; $c^{\prime }$ is the direct effect of MA on GD after controlling the mediating variable *M*. $e_{1}-e_{3}$ is the regression residual; the mediating effect is equal to the indirect effect, which is the product of the coefficients *a* and *b*.

### 4.2. Variables

#### 4.2.1. Explained Variable

Referring to Yuan et al. [47], this paper uses a DPSIR (driving force, pressure, status, impact, response) framework that includes 23 basic indicators to measure the explained variable of GD (see details in Appendix A). The entropy weight method is adopted to calculate indicator weights.

#### 4.2.2. Core Explanatory Variable

This paper uses the local entropy index to describe the level of MA. This is because the local entropy model can better eliminate the endogenous impact brought by regional-scale differences and can accurately describe the distribution of MA [48]. In the robustness test, this paper uses the Herfindahl–Hirschman Index (HHI) to remeasure the level of MA with reference to Mitchell’s research [49].

#### 4.2.3. Control Variables

Economic development level (lnEL and $\;{\left(\mathrm{lnEL}\right)}^{2}$): Measured by the linear and quadratic terms of GDP per capita. The environment Kuznets hypothesis states that with the increase in economic development, the pollutant emissions show an inverted-U trend that first increases and then reduces [50]. Thus, the linear and quadratic terms of GDP per capita are introduced in the model to describe the important impact of economic development on pollutant emissions.

Industrialization level (lnIL): Measured by the logarithm of the proportion of the added value of the secondary sector to GDP. Rapid industrialization might lead to a sharp increase in energy consumption, which would intensify pollutant emissions [51]. Therefore, this article expects this coefficient to be positive.

Industrial structure (lnIS): Measured by the logarithm of the ratio of the output value of the tertiary sector to the output value of the secondary sector. The advanced industrial structure is conducive to promoting regional environmental governance and reducing regional pollutant emissions [52]. Therefore, this article expects this coefficient to be negative.

Environmental Regulation (lnER): Measured by the logarithm of the composite index of the industrial solid waste utilization ratio, industrial soot removal ratio, and industrial sulfur dioxide removal ratio. On the one hand, local governments tend to improve environmental quality by implementing different types of environmental regulatory policies to control pollutant emissions [53]. On the other hand, because environmental regulations can lead to increased production costs for enterprises, the government may consciously lower the environmental regulatory threshold to reduce economic losses, thereby causing “racing to the bottom” among regions [54]. Therefore, it is hard to determine the sign of this coefficient.

Infrastructure (lnRD): Measured by the logarithm of the ratio of the total length of roads to the administrative area at the end of the year in each city. The level of infrastructure construction has a direct bearing on the economic development potential of a region and the choice of enterprises and has an important impact on the GD in the region. Therefore, this paper refers to Zhang et al. (2019) and uses road mileage per unit area to describe infrastructure levels [55].

#### 4.2.4. Mediating Variables

Labor force upgrading effect (lnLU): The fundamental purpose of labor force upgrading is to increase output, and the important factor that affects output increase is the education level [56]. Therefore, this paper selects the number of students per 10,000 people in higher education to measure the labor force upgrading effect.

Industrial structure upgrading effect (lnIU): The degree of industrial structure upgrading is often seen as an important indicator of the quality of a country’s economic development and its future competitiveness, which has a significant impact on economic growth and environmental quality [57]. Therefore, this paper uses the ratio of the output value of the tertiary sector to the output value of the secondary sector to describe the status of industrial structure upgrading.

Technological innovation effect (lnTI): Technological innovation can promote high-quality economic growth and environmental protection by improving technologies and processes of production. It may also exacerbate environmental pollution by increasing the scale of production [58]. This article selects urban patent entitlement per capita to describe the technological innovation effect.

### 4.3. Endogeneity and Instrumental Variables

New economic geography states that MA is endogenous to economic growth, which has significant endogenous problems by itself [59]. There are two main causes of endogeneity. First, MA can affect GD, but areas with high GD can also influence the layout of manufacturing enterprises due to the advantages of the natural environment. That said, there may be a reverse causality between GD and MA. Second, although the study controlled as comprehensive a range of factors affecting GD as possible in the model, it is still theoretically impossible to control missing variables effectively. In response to the endogeneity caused by the above factors, this paper attempts to find IVs to mitigate the estimation bias caused by endogeneity and uses the GS2SLS method that controls both the spatial spillover effect and endogeneity to estimate the parameters.

Based on the basic logic of building IVs, the IVs built in this paper should satisfy “the principle of exclusiveness”; that is, those exogenous variables that are intrinsically linked to MA only and not directly related to GD. In general, scholars tend to choose IVs from a geographical or historical perspective. Geographic IVs are often naturally exogenous, whereas historical IVs often do not have a direct impact on the explained variables, and thus, can meet exogenous requirements. On the other hand, due to natural endowments and temporal inertia, geographical or historical indicators tend to affect modern socioeconomic elements, thus satisfying the requirements for IVs. More typically, from a geographical point of view, Barone and Narciso (2015) chose relief amplitude as an IV for Italian mafia activities, analyzing the impact of organized crime on commercial subsidies [60]. From a historical perspective, Li and Lu (2009) used the urban population in 1920 and the number of industrial enterprises in 1978 as IVs for MA to analyze the impact of geographical agglomeration on vertical division of labor in Chinese manufacturing industries [61].

From an exogenous perspective, trade ports and railways are historical facts that have occurred in the past 100 years and should not affect the current GD. Moreover, relief amplitude is a natural geographical indicator. In theory, therefore, its direct impact on GD is minimal. In terms of relevance, there usually is convenient traffic, good infrastructure, and advanced education in trade ports. They are densely populated areas for commercial activities since modern times, serving as a window to the introduction of overseas capital and technology. In old China, railways played an important role in reducing the transport costs of enterprises. Relief amplitude not only directly affects the construction cost of infrastructure, but also affects population concentration, urban layout, and the location of enterprises. To summarize, these are all important factors for the formation of MA. Therefore, this paper selects whether the trade ports opened in the Qing Dynasty be-tween 1842 and 1909, whether there was a railway passing by in 1933, and relief am-plitude as IV for MA.

### 4.4. Study Area

**Figure 3:**
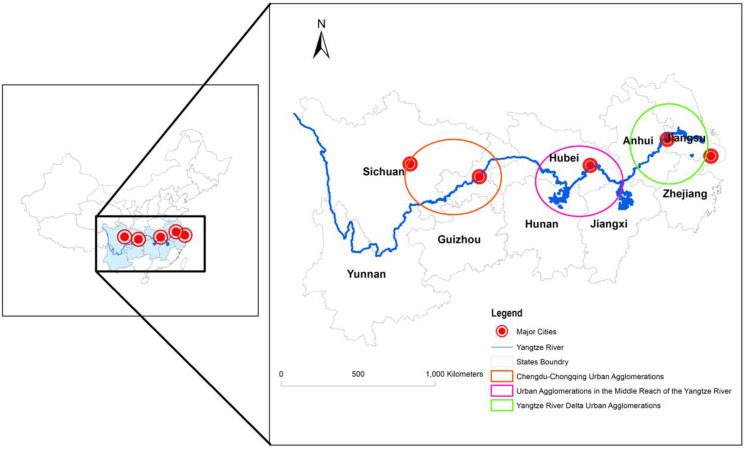
Geographical scope of the YREB.

The Yangtze River Economic Belt (YREB) covers 11 provinces and cities along the river and spans over eastern, central, and western China (Figure 3). It clusters 40% of China’s population in 20% of its land area and contributes more than 40% of China’s GDP. It has become an important national-level regional development strategy for China since 2014. Current Chinese President Xi Jinping has presided over three high-level meetings on the development of the YREB. The YREB is positioned by the Chinese government as the main battlefield for ecological priority and green development, the aorta for dual-cycle communication at home and abroad, and the main force for high-quality economic development. As a typical representative of China’s current economic and social transformation, the YREB can reflect the reality of China’s MA and GD and give an overall picture.

### 4.5. Data Source

Around 2000, there were more frequent changes in China’s urban administrative division; thus, the issue of missing data was more severe. Since 2016, the National Bureau of Statistics of China has adjusted the indicators and caliber of statistics, and no longer publishes some basic city-level indicators (GDP, total industrial production, etc.). For this reason, panel data from 110 cities in the YREB between 2003 and 2016 were selected as a research sample. Most of these economic variables are derived from the *China Urban Statistics Yearbook* and the *China Statistical Yearbook*. Patent data is derived from the China Research Data Services Platform—China Innovation Patent Research Database (https://www.cnrds.com/, accessed on 18 January 2022). Meteorological variables were collected at the National Meteorological Data Center (http://data.cma.cn/, accessed on 18 January 2022). The data about a railway passing by in 1933 were derived from the China Railway Circular, compiled by the Department of Operations of the Ministry of Railways, and Bai Shou Yi’s Edition of the *History of China Traffic* [62,63]. The trade ports opened in the Qing Dynasty between 1842–1909 were collected from a series of treaties signed between the Qing Dynasty and Western countries. Raster data of the relief amplitude were extracted using GIS technology and the geographic digital elevation model (DEM) at 1:1 million, based on a specification of 1 km × 1 km. The 3 km × 3 km grid was selected as the measurement unit. Within each measurement unit (9 km), the difference between the highest and lowest elevation was determined as the relief amplitude of each city. The DEM was derived from the Science Data Center for Dry Zones in Cold Areas of China (http://westdc.westgis.ac.cn, accessed on 18 January 2022).

To alleviate statistical bias, this paper first adds an interpolation to the individual missing data and anomalies in the dataset. Second, to remove the impact of inflation, this paper uses the year 2003 as the base period, and all price variables are adjusted using the GDP flattening index method. Third, due to the large fluctuations in long-term serial data, all variables are indented by 1% in this paper to overcome the disturbance of the empirical results from abnormal values. Finally, to reduce the possible effect of heteroscedasticity, this paper performs a logarithm on each variable. The descriptive statistics of associated variables are shown in Table 1.

To avoid the deviation of the regression results due to the collinearity between dependent variables, this paper uses the multicollinearity test and correlation coefficient test to analyze the main variables. The results show that (see details in Appendix B) the minimum value of the variance inflation factor (VIF) is 1.19 and the maximum value is 2.68, all of which are less than the threshold value of 10. This shows that there is no serious collinearity between the explanatory variables. The correlation coefficient test further confirms that the correlation coefficients between each explanatory variable are a maximum of 0.5972 and a minimum of −0.0044. The correlation coefficients between most variables pass the 10% significance test, which indicates that there is not a significantly strong or slight correlation between each explanatory variable. Therefore, the multicollinearity problem can be ignored in the regression analysis below.

## 5. Empirical Analysis

### 5.1. Spatio-Temporal Analysis of GD

#### 5.1.1. Temporal Evolution Analysis

**Figure 4:**
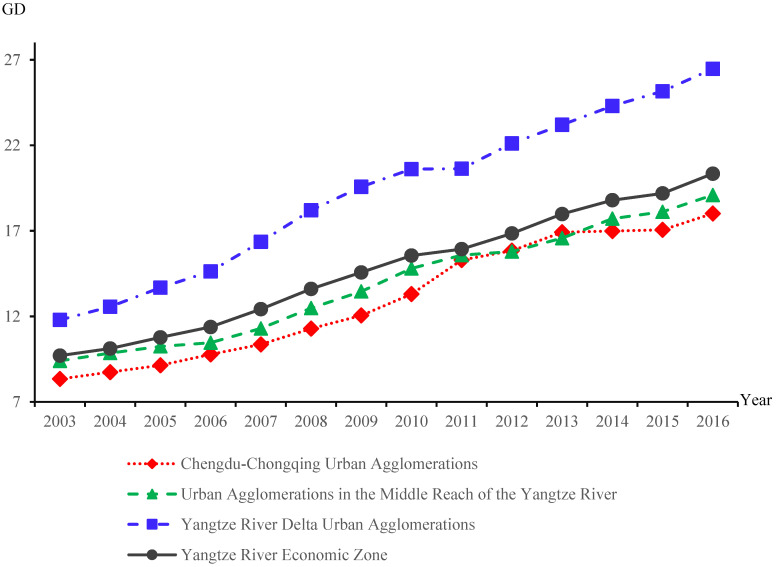
The GD of the YREB and comparison between its three major urban agglomerations.

The GD of the YREB shows an overall steady upward trend, but the differences within the three major metropolitan clusters are large and closely related to natural resource endowments. According to Figure 4, the GD of the YREB climbs from 9.704 to 20.341 between 2003 and 2016, with an average annual growth rate of 7.87%. Moreover, during the sample study period, the coefficient of variation of GD is 0.241, which indicates that the GD of the YREB has relatively little internal fluctuation. In order to further analyze the spatial and temporal distribution differences of GD in the YREB, this paper explores the issue from the perspective of urban agglomerations [64,65,66].Obviously, the GD of the Yangtze River Delta Urban Agglomerations is significantly higher than the Urban Agglomerations in the Middle Reach of the Yangtze River and Chengdu–Chongqing Urban Agglomerations. There is a huge gap between the Yangtze River Delta Urban Agglomerations and the other two urban agglomerations in terms of both total amount and speed.

#### 5.1.2. Spatial Evolution Analysis

To analyze the characteristics of spatial evolution and internal differences in the GD of the YREB, this paper uses the inverse distance weighted method to map the spatial pattern evolution of the GD of the YREB from 2003 to 2016.

**Figure 5:**
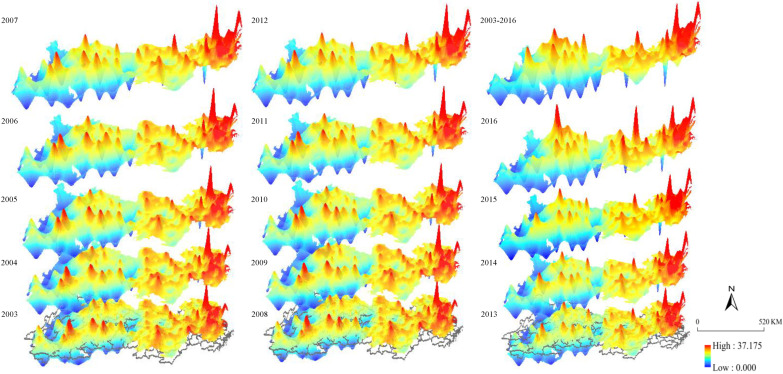
Spatial evolution pattern of the GD in the YREB.

The spatial structure of the GD of the YREB between 2003 and 2016 has clear path-dependent characteristics, and it shows a structure with “multiple peaks-multiple centers”, with very clear spatial heterogeneity. From the spatial distribution of GD between 2003 and 2016, as shown in Figure 5, we can see that the eastern region, with cities such as Shanghai, Nanjing, Suzhou, and Changzhou at its center, has gradually grown into the center of green development within the entire YREB. The central region spreads around Wuhan, and the western region with Guiyang at its center has led to an improvement in GD of the surrounding areas. This zoning difference did not fundamentally change during the entire sample study period. Although the GD of the YREB has wide spatial differences, the number and level of the “peak”s and “enter”s are rising steadily, indicating that the GD of the YREB is continuously improving.

### 5.2. Baseline Regression Result

This section starts with a spatial autocorrelation test of variables by using the global Moran’s I. The results in Appendix C show that during the sample study period, the GD, MA, EL, IL, ER, and RD are generally significant at the confidence level of 1%, indicating that relevant variables have a spatial effect. It is, therefore, necessary to include the spatial effect of variables in the model.

This paper examines the impact of MA on GD by using the OLS, FGLS, spatial generalized method of moments (GMM), and GS2SLS. According to columns (1) and (2) in Table 2, the quadratic regression coefficients of MA estimated by both the OLS and FGLS methods pass the significance test of 10%, and the linear terms pass the significance test of 1%. This indicates that there is an inverted U-shaped curve between MA and GD. However, the quadratic coefficients of MA estimated by the spatial GMM method in column (3) fail the significance test. Looking further into the GS2SLS estimates in column (4), it can be seen that the regression coefficient of MA to GD is −0.167, which is significant at the level of 10%, and the linear coefficient is −0.585, which passes the significance test of 1%. This is consistent with the results of the OLS and FGLS methods. The linear and quadratic coefficients of MA estimated by the GS2SLS method are larger than the coefficients estimated by methods of non-spatial OLS and non-spatial FGLS. This means that neglecting spatial effects and endogeneity can lead to an underestimation of the impact of MA on GD or even to a false conclusion. Therefore, the results of the subsequent analysis in this paper are based on the GS2SLS.

According to column (4), there is a significant inverted U-shaped relationship between MA and GD. This means that when MA is within a reasonable range, agglomeration brings about more positive externality than negative externality. At this point, increasing MA not only fails to increase effective economic output, but also leads to significant resource and energy consumption and pollutant emissions, thereby inhibiting an increase in GD. This conclusion is in line with the findings of Yuan et al. [8].

The possible reasons are as follows. When MA is within a reasonable range, MA can match enterprises with more specialized employees by supplying abundant labor. By extending the industrial chain through the upstream and downstream industrial links between manufacturing enterprises, MA can increase the added value of enterprises. Through exchanges and trade between enterprises, MA can promote technological spillovers and high-quality economic growth, and achieve energy savings and environmental protection, thereby increasing GD. However, when MA exceeds at a reasonable range, the inability of local infrastructure and other hardware to follow up on the huge consumption demand brought about by the concentration of large numbers of enterprises and people adversely affects the local natural environment and economic development. This is usually manifested by increased market competition, traffic congestion, and a sharp increase in pollutant emissions, resulting in an uneconomical effect of agglomeration.

In addition, the spatial lagged term for GD is 0.944, which passes the significance test of 1% and verifies that GD has significant spatial spillover effects. This means the increase in GD is influenced by the GD of surrounding cities. For every 1% increase in GD in neighboring regions, the GD of the local area can increase by 0.944%.

### 5.3. Robust Test

#### 5.3.1. Changing the Spatial Weight Matrix

This paper conducts a robust test on its core findings by using the inverse squared distance matrix and economic geographic distance matrix. As can be seen in column (1) and (2) of Table 3, MA still has a significant inverted U-shaped curve relationship with GD. This indicates that the core findings of this paper do not depend on the choice of the spatial weight matrix.

#### 5.3.2. Replacing Core Explanatory Variable

MA is the core explanatory variable of this article; therefore, the accurate measurement of MA is the key to its empirical analysis. To improve the comparability of research results, the logarithm of MA is adopted in this paper. According to column (3) in Table 3, there is still an inverted “U” curve between MA and GD. This shows that the relationship between MA and GD does not change significantly due to the different measurement methods of MA.

#### 5.3.3. Add Control Variables

The combination of a region’s meteorological factors directly determines the strength and disadvantage of its natural conditions, and the strength and potential of its economic development, which also has an important influence on its GD [67]. Therefore, this paper further controls for annual average precipitation (AAP), average wind speed (AWS), average air pressure (APR), sunshine hours (SUHs), relative humidity (RHU), and other meteorological factors, and logarithmizes each variable. It can be seen from column (3) of Table 3 that after considering the interference of meteorological factors, the linear and quadratic regression coefficients of MA are still significantly negative and are significant at the confidence levels of 5% and 1%, indicating that the inverted U-shaped curve between MA and GD would remain the same regardless of the changes in meteorological factors.

### 5.4. Heterogeneity Analysis

#### 5.4.1. Temporal Heterogeneity

Since the outbreak of the international financial crisis in 2008, China’s economy has encountered sustained downward pressure. Under this background, the Chinese government recognizes that it can no longer rely on an extensive development model with high pollution, high emissions, and high consumption, and that it must shift to a green development model with high efficiency, low consumption, and low pollution. The extensive development model before 2009 is defined as the old normality, and the green development model after 2009 is defined as the new normality [68]. Accordingly, the sample is divided into the periods of 2003–2008 and 2009–2016 to examine the impact of MA on GD from a temporal heterogeneity perspective.

According to Table 4, in the old normality period, the quadratic regression coefficient of MA is positive, but it fails the significance test, whereas the linear coefficient is significantly negative at the confidence level of 1%. This shows that MA in the old normality period inhibits the increase in GD. In the new normality period, the linear and quadratic coefficients of MA are negative, and both pass the 1% significance test. This indicates that there is an inverted U-shaped curve between MA and GD in the new normality period.

The main reason would be the economic development orientation of the Chinese government. During the old normality period, China was technologically backward, and relied mainly on the cheap investment in resources to promote economic growth. It adopted an extensive development model with high consumption, high input, high emissions, and high pollution; thus, MA impeded the improvement of GD. During the new normality period, the Chinese government has recognized that the extensive development model is unsustainable, and China must shift to a green development model with low consumption, low input, low pollution, and high efficiency, which leads to a significant flow of capital and technology to “clean” industry. However, it will take a certain amount of time to repay the ecological environmental debt owed during the historical period. Only after this period can the green effect of the MA begin to work out.

#### 5.4.2. Spatial Heterogeneity

The Yangtze River Delta Urban Agglomerations, Urban Agglomerations in the Middle Reach of the Yangtze River and Chengdu–Chongqing Urban Agglomerations are the only three nation-level urban agglomerations. They are also the core economic growth zones and key areas for improving GD in the YREB. Since the three urban agglomerations are located in different zones, the natural conditions vary greatly, and the level of economic development is uneven, as there is extremely uneven internal development among them. In this sense, this paper collected a sample of 26 cities in the Yangtze River Delta Urban Agglomerations, 31 cities in the Urban Agglomerations in the Middle Reach of the Yangtze River, and 16 cities in the Chengdu–Chongqing Urban Agglomerations according to the spatial boundaries defined in the development plans of these three urban agglomerations [69]. The GS2SLS was used to estimate the base model.

The estimated results in Table 4 show that the quadratic regression coefficients for MA in the Urban Agglomerations in the Middle Reach of the Yangtze River and Chengdu–Chongqing Urban Agglomerations pass the 5% significance test. This means that there is a significant inverted U-shaped curve between MA and GD in these two urban agglomerations. On the contrary, the quadratic coefficient for MA in the Yangtze River Delta Urban Agglomerations is significantly positive, indicating a significant positive U-shaped curve between MA and GD.

One possible reason would be the long history of the manufacturing industry in the Yangtze River Delta Urban Agglomerations. It is currently dominated by high-end manufacturing and service industries, and thus, MA promotes the increase in GD. The Urban Agglomerations in the Middle Reach of the Yangtze River are dominated by heavy industry, with huge resource consumption and a sharp increase in pollution emissions, and its agglomeration has a negative effect on GD. Constrained by natural conditions, the share of manufacturing industry in the Chengdu–Chongqing Urban Agglomerations has always been relatively low, and MA has not yet been able to play a role in promoting GD.

#### 5.4.3. Heterogeneity of Urban Characteristics

Manufacturing industries are often resource-intensive industries with high energy consumption and pollutant emissions, which have an important impact on economic growth and environmental quality. Based on the list of resource-based cities published by the State Council, the Sustainable Development Plan for National Resource-based Cities between 2013–2020 (http://www.gov.cn/zwgk/2013-12/03/content_2540070.htm, accessed on 18 January 2022), this paper selected 40 resource-based cities and 70 non-resource-based cities. It also used the GS2SLS to estimate the base model.

The regression results in Table 4 show that the linear and quadratic regression coefficients for MA in the sample of resource-based cities are significantly negative. This means that there is a significant inverted U-shaped curve between MA and GD in this sample. However, in the sample of non-resource-based cities, the quadratic coefficient for MA does not pass the significance test, whereas the linear coefficient −0.693 passes the significance test of 1%. This shows that MA in the sample of non-resource-based cities is not conducive to increasing GD.

This might have resulted from the released dividends of a scale economy by enterprises through shared resources, knowledge spillovers, and cost-sharing during the early years of MA, which have promoted green economic growth and reduced energy consumption per unit of output. The inhibition effect shown in non-resource-based cities may be due to the increase in imported pollution, the deliberate relaxation of environmental regulations by local governments to develop the economy, and the large number of investment promotion. This has led to an increase in total regional energy consumption and pollutant emissions.

## 6. Mediating Effect Test

Based on theoretical analysis, this paper further uses the mediating effect model to test the mechanism by which MA affects GD (Table 5):

The path test of “MA—labor force upgrading effect—GD”: It can be seen from column (1) of Table 5 that the linear and quadratic regression coefficients of MA are both significantly negative, indicating that there is a significant inverted U-shaped curve between MA and GD. According to column (2), there is a significant U-shaped relationship between MA and the labor force upgrading effect. As can be seen in column (3), after controlling the labor force upgrading effect in column (1), the absolute values of both linear and quadratic coefficients of MA decrease significantly. Meanwhile, the mediating effect of the labor force upgrading effect passes the Sobel test. This indicates that the labor force upgrading effect is the mechanism that causes the inverted U-shaped curve between MA and GD.

The path test of “MA—industrial structure upgrading effect—GD”: According to column (4) in Table 5, MA can promote industrial structure upgrading. According to column (5), after further controlling for the industrial structure upgrading effect, the linear and quadratic coefficients for MA decrease to −0.875 and −0.350, respectively, passing the 1% significance test. This means that the industrial structure upgrading effect is an impact path that causes the inverted U-shaped relationship between MA and GD. The mediating effect passes the Sobel significance test.

The path test of “MA—technological innovation effect—GD”: According to column (6) in Table 5, MA can promote technological innovation. After the control variable of technological innovation is added to column (1), the absolute values of the linear and quadratic regression coefficients for MA decrease significantly, but only the linear term passes the 1% significance test. A further Sobel significance test reveals that the technological innovation effect is indeed a mediating mechanism that influences the relationship between MA and GD.

## 7. Conclusions and Discussion

### 7.1. Conclusions

First, this article expands the theoretical framework of traditional industrial agglomeration by systematically analyzing the interaction mechanism between MA and GD, and by considering China’s realities. Second, based on panel data from the YREB, this paper uses the GS2SLS that can control both spatial effects and endogeneity to empirically examine the impact of MA on GD, and the heterogeneity influence of MA on GD is explored from a multidimensional perspective. Third, this paper reveals the conduction mechanism of MA affecting GD from both theoretical and empirical perspectives. The following conclusions are reached:

(1) From the perspectives of both the overall YREB and the regional differences, GD has always maintained a steady increase over time, but there are still large differences in growth rate and growth magnitude. In terms of the spatial evolution process, the entire YREB and each region show a hierarchical structure of “multiple peaks-multiple centers”. Because the areas between each peak and secondary center are continuously populated, there is a relatively large gap within each region.

(2) GD has significant spatial spillover effects, and there is an inverted U-shaped curve between MA and GD. That is, when MA is at a reasonable scale, it acts as a boost to GD, and when MA exceeds a reasonable scale, it acts as a disincentive to GD.

(3) Under the constraints of temporal, spatial, and urban heterogeneity, MA has a significantly different impact on GD. In terms of temporal heterogeneity, MA shows a clear inhibiting effect on GD in the old normality period; however, there is a significant inverted U-shaped curve between MA and GD in the new normality period. In terms of spatial heterogeneity, the relationship between MA and GD in the Urban Agglomerations in the Middle Reach of the Yangtze River and Chengdu–Chongqing Urban Agglomerations shows an inverted U-shaped curve. However, in the Yangtze River Delta Urban Agglomerations, the relationship shows a positive U-shaped curve. In terms of urban heterogeneity, MA in resource-based cities has an inverted U-shaped impact on GD, whereas MA in non-resource-based cities may hinder the improvement of GD.

(4) The industrial structure upgrading effect, labor force upgrading effect, and technological innovation effect play a mediating role between MA and GD.

### 7.2. Policy Implications

It is an urgent task for the vast number of developing countries, as it is for China, to promote the improvement of GD from the perspective of MA. Based on a systematic theoretical and empirical analysis, this paper provides the following policy recommendations for developing countries in formulating policies for MA to promote the green development transformation of their economies and societies:

(1) It is necessary to break down barriers to regional cooperation and bring into play the regional linkage effect. First, efforts need to be taken to remove barriers of cooperation caused by administrative boundaries, local protections, and other factors to allow for the free movement of resources between cities and to improve the efficiency of resource allocation. Second, the key to breaking up barriers of cooperation lies in rational distribution of benefits. If the government wants to promote regional cooperation on a broader scale, it must pay attention to the use of tax, fiscal, and other economic instruments. Appropriate subsidies should be provided to areas where interest is impaired so that economic dividends and green benefits are shared among the collaborators in the region.

(2) It is necessary to formulate differentiated MA policies and avoid “one size fits all”. In line with the dynamic changes in the level of MA, it is necessary to adjust relevant regional policies in a timely manner, eliminate non-economic effects of agglomeration, and bring into play the economic effect of agglomeration. On the one hand, for areas where MA is within a reasonable range, it is necessary to not only properly control the degree of MA to avoid crowding effects, but also actively guide MA to transform from increasing quantitatively to developing qualitatively. On the other hand, for regions where MA is excessive, it is necessary to not only eliminate backward enterprises by means of market economy and policies, but also attract high-quality enterprises with low pollution and high added value, thereby optimizing the industrial structure within the concentration area.

(3) It is necessary to form a situation coordinated by technological innovation, industrial structural upgrading, and labor force upgrading. First, it is necessary to encourage and support the free flow of information, personnel, and other elements within the concentrated areas to introduce advanced technologies and to promote domestic enterprises to innovate in technology. Second, the continuation of agglomeration is necessary for promoting industrial structure upgrading and labor force upgrading, and for raising the threshold for industries. Third, through training in vocational skills and the use of big data, the quality of the labor force can be improved and the mismatch between enterprises and employees can be reduced.

### 7.3. Critical Analysis and Discussion

The possible limitations of this paper and the directions of future studies are as follows:

(1) Although this paper used panel data from 110 cities in the YREB of China to conduct targeted and heterogeneous analyses, samples from other regions or countries are not considered. Therefore, future research can use samples from multiple countries to conduct in-depth comparative analyses by taking into account heterogeneity factors such as different regions, different income levels, and different urbanization stages.

(2) Although this paper systematically analyzed the impact of MA on GD, it does not explore the impact of other types of industrial agglomeration on GD. Therefore, future research can analyze the impact and mechanism of different types of industrial agglomeration, such as financial agglomeration and service industry agglomeration on GD, to improve the level of green development more comprehensively.

## Figures and Tables

**Table 1:** Descriptive statistics of variables.

Variables	Definition	Sample Size	Mean	Std. Dev	Min	Max	Unit
lnGD	Comprehensive evaluation of DPSIR model	1540	2.626	0.367	1.807	3.616	-
lnMA	Location quotient index	1540	−0.231	0.57	−2.052	0.859	-
lnEL	Per capita GDP	1540	9.728	0.839	7.926	11.749	Yuan per capita
lnIL	Proportion of added value of the secondary sector to GDP	1540	−5.326	0.237	−6.211	−4.89	%
lnIS	Ratio of the output value of the tertiary sector to the output value of the secondary sector	1540	−0.261	0.345	−1.043	0.756	%
lnER	Composite index	1540	−0.271	0.193	−0.978	−0.039	-
lnRD	Ratio of the total length of roads to the area of the administrative area at the end of the year	1540	0.713	0.929	−1.54	2.646	%
lnLU	Number of students per 10,000 people in higher education	1540	5.874	0.386	5.461	7.235	Persons
lnIU	Ratio of the output value of the tertiary sector to the output value of the secondary sector	1540	−0.261	0.345	−1.043	0.756	%
lnTI	Urban patent entitlement per capita	1540	0.139	1.979	−6.08	4.162	Items

**Table 2:** Estimation results of impact of MA on GD.

Variables	OLS	FGLS	Spatial GMM	GS2SLS
	(1)	(2)	(3)	(4)
lnMA	−0.132 ***	−0.109 ***	−0.088 ***	−0.585 ***
	(−9.83)	(−10.62)	(−5.93)	(−7.14)
(lnMA)^2^	−0.0220 **	−0.012 *	−0.010	−0.167 *
	(−2.43)	(−1.73)	(−1.03)	(−1.74)
lnEL	−0.545 ***	−0.531 ***	−0.492 ***	−0.531 ***
	(−5.64)	(−6.92)	(−5.10)	(−4.24)
(lnEL)^2^	0.041 ***	0.040 ***	0.038 ***	0.039 ***
	(8.33)	(10.44)	(7.84)	(6.13)
lnIL	−0.101 ***	−0.119 ***	−0.102 ***	0.064
	(−4.92)	(−6.94)	(−4.53)	(1.43)
lnER	0.725 ***	0.767 ***	0.762 ***	0.685 ***
	(26.88)	(34.42)	(28.22)	(20.45)
lnRD	0.063 ***	0.036 ***	0.057 ***	0.031 **
	(8.83)	(6.31)	(6.71)	(2.06)
W*lnGD				0.944 ***
				(10.70)
Constant	3.646 ***	3.464 ***	3.365 ***	3.965 ***
	(6.98)	(8.20)	(6.41)	(5.35)
City fixed effect	Yes	Yes	Yes	Yes
Adjusted R^2^	0.821			0.991
Hausman test				19.009 ***
Sample size	1540	1540	1540	1540
Inflection point	0.050	0.011	0.012	0.174

Note: ***, **, * indicate significance at confidence levels of 1%, 5%, and 10%, respectively, and the data in parenthesis are t-statistics.

**Table 3:** Estimation results of robust test.

Variables	Inverse Squared Distance Matrix	Economic Geographic Distance Matrix	Replacing Core Explanatory Variable	Increasing Control Variables
	(1)	(2)	(3)	(4)
lnMA	−0.771 ***	−1.057 ***	−0.582 **	−0.339 ***
	(−6.58)	(−4.67)	(−2.28)	(−5.43)
(lnMA)^2^	−0.315 **	−0.519 **	−0.020 **	−0.123 **
	(−2.12)	(−2.27)	(−2.23)	(−2.05)
lnHHI			−0.582 **	−0.339 ***
			(−2.28)	(−5.43)
(lnHHI)^2^			−0.020 **	−0.123 **
			(−2.23)	(−2.05)
W*lnGD	132.613 ***	0.000 ***	0.875 ***	1.131 ***
	(8.39)	(3.99)	(5.89)	(13.49)
Constant	2.890 ***	1.887	0.247	12.496 ***
	(3.15)	(1.23)	(0.17)	(6.40)
Control variables	Yes	Yes	Yes	Yes
Meteorological factors	Yes	Yes	Yes	Yes
Adjusted R^2^	0.9887	0.9861	0.9876	0.9946
Sample size	1540	1540	1540	1540

Note: ***, **, * indicate significance at confidence levels of 1%, 5%, and 10%, respectively.

**Table 4:** Results of heterogeneity analysis.

Variables	Old Normality (2003–2008)	New Normality (2009–2016)	Yangtze River Delta Urban Agglomerations	Urban Agglomerations in the Middle Reach of the Yangtze River	Chengdu–Chongqing Urban Agglomerations	Resource-Based Cities	Non-Resource-Based Cities
	(1)	(2)	(1)	(2)	(3)	(1)	(2)
lnMA	−0.355 ***	−0.248 **	−0.359 ***	−0.145	−1.624 **	−0.851 ***	−0.693 ***
	(−3.97)	(−2.55)	(−3.80)	(−0.72)	(−2.46)	(−4.04)	(−6.36)
(lnMA)^2^	0.018	−0.345 ***	0.926 *	−0.627 **	−1.134 **	−0.495 ***	−0.175
	(0.15)	(−4.94)	(1.83)	(−1.99)	(−2.23)	(−3.17)	(−1.39)
lnEL	−1.626 ***	−0.657 ***	0.447	−0.441	−0.026	−0.643 **	−0.298 *
	(−6.45)	(−4.10)	(0.73)	(−1.50)	(−0.02)	(−2.03)	(−1.90)
(lnEL)^2^	0.104 ***	0.044 ***	−0.005	0.034 **	0.013	0.045 ***	0.025 ***
	(7.92)	(5.57)	(−0.15)	(2.27)	(0.23)	(2.82)	(3.11)
lnIL	−0.013	−0.186 **	−0.272	−0.014	−0.097	−0.022	0.159 **
	(−0.25)	(−2.12)	(−1.44)	(−0.22)	(−0.34)	(−0.29)	(2.15)
lnER	0.580 ***	0.509 ***	1.109 ***	0.831 ***	0.648 ***	0.733 ***	0.698 ***
	(15.75)	(8.63)	(5.48)	(9.53)	(2.97)	(10.53)	(14.82)
lnRD	0.046 **	−0.038 *	−0.019	−0.060	0.002	0.019	0.055 **
	(2.49)	(−1.71)	(−0.54)	(−1.30)	(0.02)	(0.59)	(2.54)
W*lnGD	0.173	0.534 ***	0.707	1.453 ***	−0.410	2.467 ***	1.547 ***
	(1.46)	(4.31)	(1.52)	(4.27)	(−0.49)	(4.36)	(7.45)
Constant	8.422 ***	3.776 ***	−2.903	3.410 **	1.276	4.031 **	3.462 ***
	(6.16)	(4.03)	(−0.75)	(2.26)	(0.20)	(2.36)	(3.65)
Adjusted R^2^	0.9920	0.9928	0.9915	0.9941	0.9555	0.9837	0.9901
Sample size	660	880	364	392	224	560	980
Inflection point	-	0.698	1.214	0.891	0.489	0.423	-

Note: ***, **, * indicate significance at confidence levels of 1%, 5%, and 10%, respectively.

**Table 5:** Impact path tests.

Variables	Total Effect	Labor Force Upgrading Effect		Industrial Structure Upgrading Effect		Technical Innovation Effect	
	lnGD	lnLU	lnGD	lnIU	lnGD	lnTI	lnGD
	(1)	(2)	(3)	(4)	(5)	(6)	(7)
lnMA	−0.884 ***	0.551 ***	−0.662 ***	0.249 **	−0.875 ***	2.105 ***	−0.655 ***
	(−7.50)	(2.82)	(−7.59)	(2.34)	(−7.70)	(3.25)	(−7.07)
(lnMA)^2^	−0.395 ***	0.476 ***	−0.164 *	0.054	−0.350 ***	0.480	−0.094
	(−3.12)	(3.80)	(−1.72)	(0.54)	(−2.89)	(0.87)	(−0.96)
lnLU			0.026				
			(0.68)				
lnIU					0.056		
					(1.52)		
lnTI							0.047 ***
							(6.01)
lnEL	−0.474 ***	−0.917 ***	−0.549 ***	−0.909 ***	−0.457 ***	1.183 **	−0.564 ***
	(−3.04)	(−6.88)	(−3.97)	(−8.81)	(−2.87)	(2.36)	(−4.15)
(lnEL)^2^	0.036 ***	0.053 ***	0.039 ***	0.048 ***	0.035 ***	−0.036	0.038 ***
	(4.54)	(7.54)	(5.60)	(9.22)	(4.33)	(−1.39)	(5.48)
lnIL	0.107 *	0.022	0.104 **	−0.929 ***	0.188 ***	−0.303 *	0.119 **
	(1.89)	(0.51)	(2.12)	(−21.57)	(2.63)	(−1.80)	(2.44)
lnER	0.685 ***	−0.027	0.684 ***	−0.094 ***	0.691 ***	0.113	0.636 ***
	(16.45)	(−0.88)	(18.90)	(−3.65)	(16.55)	(0.79)	(16.87)
lnRD	0.036 *	0.066 ***	0.038 **	−0.017	0.041 **	0.391 ***	0.024
	(1.92)	(5.02)	(2.28)	(−1.42)	(2.21)	(7.19)	(1.48)
W*lnGD	0.936 ***		0.951 ***		0.936 ***		0.760 ***
	(8.60)		(10.16)		(8.74)		(7.11)
W*lnLU		0.022					
		(0.18)					
W*lnIU				2.878 ***			
				(16.46)			
W*lnTI		0.022				2.830 ***	
		(0.18)				(22.75)	
Constant	3.936 ***	9.753 ***	4.104 ***	−0.784	4.285 ***	−9.730 ***	4.735 ***
	(4.27)	(13.40)	(4.66)	(−1.31)	(4.68)	(−3.50)	(5.86)
Sobel test (*p*-value)		−2.197 (0.028)		−2.077 (0.038)		3.846 (0.000)	
City fixed effect	Yes	Yes	Yes	Yes	Yes	Yes	Yes
Sample size	1540	1540	1540	1540	1540	1540	1540

Note: ***, **, * indicate significance at confidence levels of 1%, 5%, and 10%, respectively.

## Data Availability

The data in this paper come from the *China City Statistical Yearbook* and the China Economic Net Statistical Database.

## Abbreviations

MA	Manufacturing agglomeration
GD	Green development
YREB	Yangtze River Economic Belt
GS2SLS	Generalized spatial two-stage least squares
GDP	Gross domestic product
HHI	Herfindahl–Hirschman index
IV	Instrumental variable
DEM	Digital elevation model
GIS	Geoinformation system
VIF	Variance inflation factor
OLS	Ordinary least squares
FGLS	Feasible generalized least squares

## Appendix A

**Table A1:** DPSIR Indicator System of GD.

Criterion	Basic Indicators	Units	Attributes
Driving force of green development (D)	Labor productivity in the primary sector	10^4^ yuan/person	Positive
	Labor productivity in the secondary sector	Ten thousand yuan/person	Positive
	Labor productivity in the tertiary sector	10^4^ yuan/person	Positive
	The percentage of science and technology expenditure in local public expenditure in the city	%	Positive
Pressure of green development (P)	The proportion of added value of the tertiary sector	%	Positive
	Industrial sulfur dioxide emissions per GDP	Ton/10^4^ yuan	Negative
	Industrial soot emissions per GDP	Ton/10^4^ yuan	Negative
	Industrial wastewater emissions per GDP	Ton/yuan	Negative
	Energy consumption per unit of gross regional product	Kilowatt hour/yuan	Negative
Status of green development (S)	Industrial sulfur dioxide emissions per capita	Ton/person	Negative
	Industrial sulfur dioxide emissions per capita	Ton/person	Negative
	Industrial wastewater emissions per capita	10^4^ tons/person	Negative
	The proportion of the number of employees in manufacturing industry to the year-end number of employees per unit	%	Negative
Impact of green development (I)	The year-end balance of savings for urban and rural residents	10^4^ yuan	Positive
	Teacher–student ratio in general primary schools	People/10^4^ people	Positive
	Teacher–student ratio in general middle schools	People/10^4^ people	Positive
	Green coverage of the completed area	%	Positive
	Green area in park per person	Square meters/person	Positive
	Green area in city per person	Square meters/person	Positive
Response of green development (R)	Industrial sulfur dioxide removal ratio	%	Positive
	Industrial soot removal ratio	%	Positive
	Industrial solid waste utilization ratio	%	Positive
	Domestic sewage treatment ratio	%	Positive
	Harmless treatment ratio of domestic garbage	%	Positive

## Appendix B

**Table A2:** Multicollinearity Tests and Correlation Coefficient between Variables.

Variables	VIF	lnGD	lnMA	lnEL	lnIND	lnER	lnRD	lnLU	lnIS	lnTI
lnGD	-	1.000								
lnMA	1.87	0.290 *	1.000							
lnEL	3.89	0.836 *	0.497 *	1.000						
lnIND	2.92	0.115 *	0.496 *	0.263 *	1.000					
lnER	1.82	0.746 *	0.315 *	0.636 *	0.237 *	1.000				
lnRD	4.05	0.681 *	0.552 *	0.776 *	0.318 *	0.503 *	1.000			
lnLU	2.07	0.554 *	0.332 *	0.609 *	0.130 *	0.327 *	0.686 *	1.000		
lnIU	2.62	0.016	−0.355 *	−0.099 *	−0.764 *	−0.177 *	−0.120 *	0.069 *	1.000	
lnTI	4.01	0.732 *	0.563 *	0.808 *	0.277 *	0.602 *	0.797 *	0.567 *	−0.128 *	1.0000

Note: ***, **, * indicate significance at confidence levels of 1%, 5%, and 10%, respectively.

## Appendix C

**Table A3:** Global Moran’s I Statistics and Their Significance.

Year	lnGD	lnMA	lnEL	lnIL	lnER	lnRD
2003	0.058 ***	0.090 ***	0.202	0.046 ***	0.088 ***	0.142 ***
2004	0.084 ***	0.115 ***	0.203	0.043 ***	0.074 ***	0.150 ***
2005	0.105 ***	0.115 ***	0.204	0.031 ***	0.090 ***	0.154 ***
2006	0.117 ***	0.115 ***	0.205	0.018 **	0.085 ***	0.150 ***
2007	0.143 ***	0.131 ***	0.205	0.010 *	0.087 ***	0.147 ***
2008	0.175 ***	0.131 ***	0.205	0.004	0.094 ***	0.131 ***
2009	0.187 ***	0.136 ***	0.207	0.006	0.072 ***	0.177 ***
2010	0.175 ***	0.135 ***	0.202	0.002	0.072 ***	0.181 ***
2011	0.112 ***	0.122 ***	0.089	0.003	0.049 ***	0.111 ***
2012	0.154 ***	0.129 ***	0.182	0.004	0.045 ***	0.174 ***
2013	0.133 ***	0.113 ***	0.162	0.006	0.049 ***	0.170 ***
2014	0.136 ***	0.127 ***	0.160	0.008 *	0.086 ***	0.158 ***
2015	0.141 ***	0.131 ***	0.158	0.016 **	0.073 ***	0.160 ***
2016	0.139 ***	0.141 ***	0.155	0.011 **	0.093 ***	0.150 ***

Note: ***, **, * indicate significance at confidence levels of 1%, 5%, and 10%, respectively.
